# *Astragalus* polysaccharide protects sepsis model rats after cecum ligation and puncture

**DOI:** 10.3389/fbioe.2022.1020300

**Published:** 2022-10-20

**Authors:** Jun Li, Jie Zhao, Yihui Chai, Wen Li, Xiaoqing Liu, Yunzhi Chen

**Affiliations:** ^1^ Resource Institute for Chinese & Ethnic Materia Medica, Guizhou University of Traditional Chinese Medicine, Guiyang, China; ^2^ School of Basic Medicine, Guizhou University of Traditional Chinese Medicine, Guiyang, China; ^3^ School of Marxism, Guizhou University of Traditional Chinese Medicine, Guiyang, China

**Keywords:** *Astragalus* polysacharin, inflammatory factors, short-chain fatty acids, vitamin D axis, corticosterone, intestinal flora, sepsis

## Abstract

To investigate the protective effect and mechanism of *Astragalus* polysaccharide (APS) on septic rats, the present project applied APS at concentrations of 400, 600, and 800 mg/kg/d to rats for prophylactic administration for 7 d, and a rat sepsis model was constructed by the cecum ligation and puncture (CLP) method. Forty-eight rats were divided into six groups of eight each. Each experiment was repeated at least three times. Rat serum levels of VD_3_, 25(OH)D_3_, 1,25(OH)_2_D_3_, IL-6, TNF-α, CRP, sICAM-1, corticosterone (CORT), and short-chain fatty acids (SCFAs) in each group were detected, and renal damage was observed by H&E. We also determined the protein expression of CYP27B1, CYP24A1, vitamin D receptor (VDR), steroidogenic acute regulatory protein (STAR), 3β-hydroxysteroid dehydrogenase (3β-HSD), CYP21A2, CYP17A1, and CYP11B1. An operational taxonomic unit (OTU) was used to determine the gut microbiota diversity of septic rats after prophylactic administration and before modeling. Results revealed that APS markedly increased the contents of 25(OH)D_3_ and 1,25(OH)_2_D_3_ but greatly decreased those of TNF-α, IL-6, CRP, sICAM-1, and CORT. APS alleviated renal tubular dilation and vascular congestion in rat kidneys and substantially reduced renal cell apoptosis. Moreover, the expression of CYP24A1, VDR, CYP11B1, CYP21A2, CYP17A1, STAR, and 3β-HSD in the kidneys of the H-APS group was substantially decreased compared to that of the model group, whereas CYP27B1 was markedly increased. GC-MS detection indicated a substantial increase in SCFAs and acetic acid content in the H-APS group versus model group. Through 16S sequencing, the abundance of genus and gut microbiota species increased in the APS groups compared to that of the control group. Taken together, APS increased the activity of the vitamin D axis, inhibited the production of inflammatory factors in the body, altered the structure of rat intestinal flora, and increased the amount of acetic acid and SCFAs in rats, thereby effectively hindering inflammation and organ damage in septic rats.

## Graphical Abstract

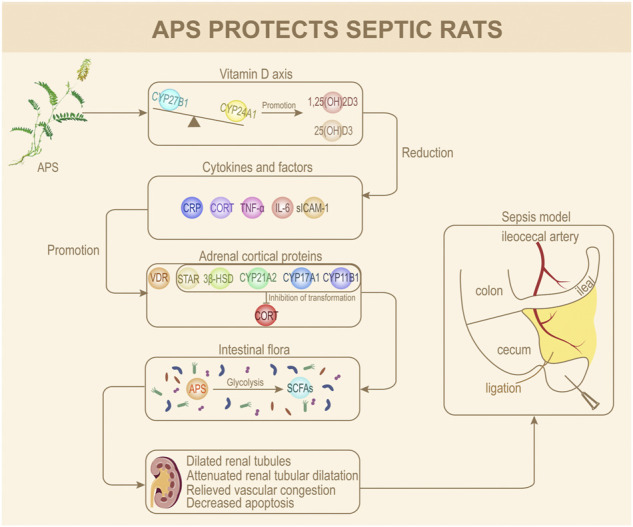

## Introduction

Sepsis is caused by an abnormal host response to infection, and acute organ dysfunction can occur in severe cases. It can be induced by a variety of microorganisms. Clinical symptoms include definite foci of infection and typical continuous or remittent fever, also with the presence of rapid breathing, decreased blood pressure, and weak but fast pulses (Singer et al., 2016). Statistically, the global incidence of sepsis is 43.7 in 10,000 individuals, of which an estimated 70% of them are complicated by encephalopathy, 47.5% by kidney injury, and 37.3% by septic shock, with an annual mortality of 40% (Machado et al., 2017; Rudd et al., 2020). With this in mind, the World Health Organization has made sepsis a priority for global disease control. Lately, management of sepsis mortality reduction often applies antibiotics combined with body fluid resuscitation, but problems such as appropriate administration dosage, unsatisfactory efficacy, and high risk of secondary diseases are also present (Guo et al., 2021). Therefore, the discovery of effective prevention methods and underlying treatment mechanisms for sepsis is urgent and critical to global health issues to date. In addition to the characteristics of fewer side effects, traditional Chinese medicine for sepsis is also significantly better than the aforementioned treatments in terms of relieving systemic symptoms, regulating multiple systems of the body, and improving prognosis (Zhao et al., 2017; Zheng et al., 2019).

*Astragalus* (Chinese: Huang qi) is commonly used as a single herb for sepsis in clinics. It has the function of nourishing people’s vitality, promoting the secretion of saliva, and enriching the blood. Its benefits also include securing the exterior, disinhibiting water and dispersing swelling, and promoting pus discharge and tissue regeneration. Its main active ingredient, *Astragalus* polysaccharide (APS), has anti-stress, antioxidant, antiviral, immune-balancing, and anti-inflammatory effects (Wu et al., 2017). Studies have confirmed that APS can inhibit mitochondrial oxidative stress damage, promote dendritic cells to differentiate into CD11c^low^CD45RB^high^ phenotype monocytes, and upregulate Th1/Th2 cytokines and the CD4+/CD8+ ratio in sepsis. Meanwhile, it can also reduce the content of TNF-α, IL-β, and IL-6 in the body and activate the AMPK/SIRT1 signaling pathway to reduce tissue damage and apoptosis caused by a systemic immune inflammatory response, thereby protecting ventricular pumping function and renal metabolism function and preventing the occurrence and development of microcirculation disorders (Yan et al., 2011; Wang et al., 2021). In general, APS in the treatment of sepsis has advantages in two-way immune regulation, inhibition of inflammatory response, enhancement of immune response, and maintenance of microcirculatory homeostasis, allowing us to effectively alleviate organ damage caused by the occurrence of sepsis and minimization of complex shock. However, those studies have not yet elucidated how APS is metabolized *in vivo* to exert its antiseptic effect.

Polysaccharides enter the digestive system and are converted into small molecular substances called short-chain fatty acids (SCFAs) by gut microbiota, reach the blood and then various parts of the body after transportation, and exert effects by binding to the targets (Layden et al., 2013). As SCFAs can affect the release of inflammatory factors, immune chemotactic responses, and proliferation inhibition of immune effector cells in sepsis (Valdés-Duque et al., 2020), it is speculated that SCFAs function as the active substance of APS against sepsis. Taken together, the present study established a sepsis rat model by observing the prophylactic administration of APS using a CLP method and explored the effects of APS on the kidneys, vitamin D axis, adrenal cortex, SCFAs, and intestinal flora of the sepsis model rat after entering the body. Subsequently, the underlying mechanisms of APS in the prophylaxis and cure of sepsis were clarified, which may provide a potential therapeutic approach for sepsis using traditional Chinese medicine administration.

## Materials and methods

### Animal grouping and prophylactic administration

Forty-eight 12-week-old male SD rats, weighing 220–320 g, were supplied by Changsha Tianqin Biotechnology Co., Ltd. with the laboratory animal license number: Scxk (Xiang) 2019–0013. Animals were allowed to eat and drink freely. The temperature and humidity of the animal housed were at 23 ± 2°C and 50% ± 5%, respectively. The light/dark cycle was 12:12 h. The rats were weighed and divided into the control group, sham operation group, model group, low-dose APS group (L-APS), medium-dose APS group (M-APS), and high-dose APS group (H-APS), with eight rats in each group. Of these, the first three groups were intragastrically gavaged with 1 ml/100 g of normal saline, and the latter treatment groups were given 400, 600, and 800 mg/kg/d APS, respectively, for 7 consecutive days.

### Establishment of a sepsis rat model by cecum ligation and puncture

The rats were fasted for 12 h and weighed before the model establishment. Except for the control group, each rat was anesthetized with 2% sodium pentobarbital (0.3 ml/100 g) by abdominal cavity injection. The rats in the model, L-APS, M-APS, and H-APS groups were used to establish the sepsis model *via* the CLP method. The detailed surgical procedures were as follows: the hair of the median abdomen of the rats was shaved and disinfected with iodine and medical alcohol. The abdominal cavity was incised about 2 cm along the linea alba to locate the cecum, which was ligated at about 1/3 of the cecum using a 5–0 suture. The cecum was stabbed using a needle and ligated twice. A small number of intestinal contents were extruded to escape out of the puncture hole to ensure the cecum was unobstructed. The treated cecum was reintroduced into the peritoneal cavity, and the inner layer was sutured using the 5–0 thread, and the outer layer, with 3–0 thread. Finally, the rats were rested on a heating mat to keep the rectal temperature at 37 ± 0.5°C. After the rats were awake, they were fed normally and had free access to water. The sham operation group performed the same procedures excluding cecal ligation and puncture.

### ELISA detection of the contents of VD3, 25(OH)D_3_, 1,25(OH)_2_D_3_, IL-6, TNF-α, CRP, sICAM-1, and corticosterone in the serum of rats

Here, 24 h after surgery, 2% sodium pentobarbital (0.3 ml/100 g) was injected into the rats in each group for anesthetization. The blood was sampled from the ventral aorta and left undisturbed at 25°C for 1 h, and centrifugation was performed to collect the serum. The content of VD3, 25(OH)D_3_, and 1,25(OH)2D3 was determined as per instructions of the ELISA kit, and the absorbance was determined at 450 nm using a spectrophotometer.

### H&E and TUNEL staining assessment of renal tissue damage

The rats were euthanized after blood collection, and kidney tissues were obtained, rinsed using normal saline, and fixed with 4% paraformaldehyde for 24 h. Subsequently, HE staining and fluorescence TUNEL staining of the kidneys were performed to evaluate the renal tissue damage. Images were acquired using a Pannoramic 250 digital slide scanner (3DHISTECH, Hungary). The calculation formula of apoptotic cells was apoptosis rate % = apoptotic cells/(normal cells + apoptotic cells).

### Immunofluorescence double staining detection of renal CYP27B1 and CYP24A1 protein expression

The rat kidney tissue was frozen and sliced with a cryomicrotome. The sections were rinsed three times using pre-cooled PBS for 5 min each time and then fixed with 4% paraformaldehyde at 25°C for 15 min. Next, 0.5% Triton X-100 was added dropwise and incubated for 15 min at 25°C. Following three cycles of washing with PBS, 5 min each cycle, the primary antibody was supplemented and incubated overnight at 4°C. The sections were taken out the next day. After repeated washing, the secondary antibody was added for light avoidance incubation for 1 h at 25°C. After PBS washing again, one drop of the anti-quenching fluorescent mounting medium was supplied and the sections were sealed. Images were acquired using a confocal fluorescence microscope.

### Western blotting detection of the protein expression of VDR, STAR, 3β-HSD, CYP21A2, CYP17A1, and CYP11B1 in the adrenal cortex

The total protein of the rat adrenal gland was lysed using RIPA lysate. Of 50 μL, the protein sample was taken, added with 5× loading buffer at a ratio of 4:1, mixed well, and denatured using a thermocycler at 95°C for 15 min. Then, electrophoresis using 10% SDS-PAGE was performed. Protein transfer was performed using PVDF at 200 mA for 1–2 h. After transfer was completed, the PVDF membrane was placed into 5% skim milk diluted with TBST and incubated on the shaker for 2 h. The primary antibody was added to the membrane for incubation overnight at 4°C. The secondary antibody was subsequently supplemented for incubation at 25°C for 2–3 h, and the PVDF membrane was washed three times with TBST. The primary antibodies 3β-HSD (BS-3906r, 1:2000) and CYP11B1 (bs-3898r, 1:2000) were purchased from Bioss Biotechnology, Beijing, China; CYP17A1 (14447-1-ap, 1:1000), STAR (12225-1-ap, 1:2000), and VDR (67192-1-lg, 1:2000) were bought from Proteintech, Chicago, United States; CYP21A2 (#C04110H, 1:1000) was purchased from Santa Cruz Biotechnology, Texas, United States; and β-tubulin (AC021, 1:5000) was bought from ABclonal, Wuhan, China. The secondary antibody Goat Anti-Mouse IgG (ab6789, 1:5000) was supplied by Abcam, Boston, United States, and Goat Anti-Rabbit IgG (S0001, 1:5000) was purchased from Affinity Biosciences, Ohio, United States. Finally, development was carried out using the ECL luminescent solution, the bands were exposed and scanned by Tanon GIS chassis control software V2.0, and gray values were measured by ImageJ software.

### GC-MS-based targeted metabolomics analysis of short-chain fatty acids in rat serum

The standard substances of acetic acid, propionic acid, butyric acid, isobutyric acid, isovaleric acid, and hexanoic acid were first prepared to standard concentration gradients of 0.1, 0.5, 1, 5, 10, 20, 50, and 100 μg/ml, respectively, using ethyl acetate. Of 600 μl, the standard product was added to 25 μl of 4-methyl valeric acid as the internal reference, then transferred into a sample vial, and loaded to GC-MS for detection. Then, 30 mg of serum samples were transferred to a 2-ml EP tube, supplemented with 900 μl of 0.5% phosphoric acid, resuspended, and mixed well by vibration for 2 min. The samples were centrifuged, and 800 μl of the supernatant was collected, extracted with an equal amount of ethyl acetate, blended well by vibration, and centrifuged at 14,000 g for 10 min. Of 600 μl, the upper organic phase was collected for GC-MS for detection, as described previously. The gas chromatography conditions were set as follows: the initial temperature was set at 90°C, increased to 120°C at 10°C/min, then increased to 150°C at 5°C/min and 250°C at 25°C/min, and maintained for 2 min. Helium was the carrier gas at a flow rate of 1.0 ml/min. The mass chromatography conditions were set as follows: the inlet temperature was 250°C, the ion source temperature was 230°C, the transfer line temperature was 250°C, and the quadrupole temperature was 150°C. Chromatographic peak areas and retention were detected by MSD ChemStation software. A standard curve was plotted, and the SCFA level in the samples was calculated.

### Diversity detection of intestinal flora in rats by 16S sequencing

After 24 h of APS prophylactic administration, the excrement of rats in control, L-APS, M-APS, and H-APS groups was collected. First, the workbench was wiped using alcohol, the rats were induced to defecate through abdominal massage, and two to three grains of feces were collected from each rat in labeled sterile cryotubes. After collection, the cryotubes were placed in liquid nitrogen for quick freezing for 1 h, sealed, and preserved in a −80°C refrigerator. Total DNA samples were extracted according to the instructions of the DNA extraction kit. After the PCR products were quantified, the official Illumina linker sequence was added to the outer end of the target region *via* PCR amplification. Following being eluted using Tris-HCl buffer, the products were detected by electrophoresis and denatured with NaOH, and a single-stranded DNA fragment was generated for MiSeq library construction. The Illumina MiSeq PE300 platform was used to obtain the base sequence information of the bacterial 16S rRNA gene in the V3-V4 variable region. QIIME software was used for quality control and OTU clustering on the obtained sequences, and the taxa (phylum, class, order, family, genus, and species) and abundance of OTU were obtained. The abundance and uniformity of gut microbiota in the samples were analyzed using alpha diversity. LEfSe was applied to identify multi-level species difference discrimination and pairwise comparison of differences, and the abundance differences in the intestinal microbial community of rats in each group were analyzed.

### Statistical analysis

Statistical analysis was conducted on the experimental data by SPSS 20.0 software. If the data obtained from each group conformed to normality and homogeneity of variance, the one-way variance method was adopted for analysis. After a satisfactory statistical significance was reached from the one-way variance analysis, the LSD method was used for comparison. Otherwise, the rank-sum test method was used for comparison. *p* < 0.05 was considered a statistically significant difference.

## Results

### Effect of *Astragalus* polysaccharide on serum levels of VD3, 25(OH)D_3_, 1,25(OH)_2_D_3_, IL-6, TNF-α, CRP, CORT, and sICAM-1 in septic rats

**FIGURE 1:**
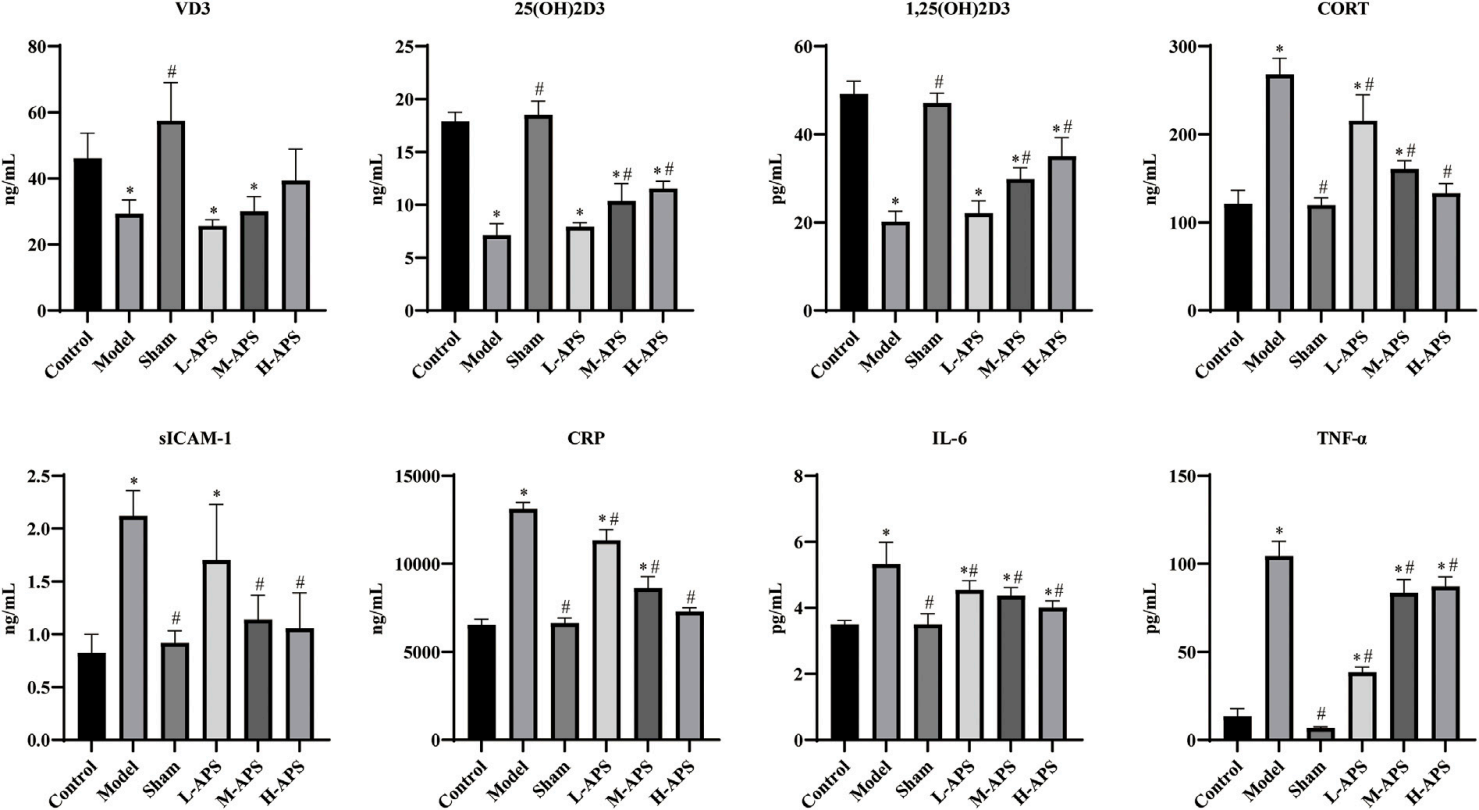
ELISA detection of the contents of VD_3_,25(OH)D_3_, 1,25(OH)_2_D_3_, IL-6, TNF-α, CRP, SICAM-1, and CORT in the serum. *p < 0.05 vs. control; ^#^ *p* < 0.05 vs. model.

After the sepsis model, rats were intervened with different doses of APS, and the serum was obtained for the ELISA assay. As compared to the model group, the VD_3_ level of each APS group had no significant difference, but the VD_3_ content had an increasing trend with the increase of APS concentration. The contents of 25(OH)D_3_ and 1,25(OH)2D3 in the H-APS group were markedly higher than those in the model group. After the sepsis model was established using the CLP method, the serum contents of CORT, IL-6, TNF-α, CRP, and sICAM-1 in the model group were substantially increased compared to those of the control group. Compared with the model group, the M- and H-APS groups could largely reduce the contents of CORT CRP, sICAM-1, IL-6, and TNF-α in rats (Figure 1).

### *Astragalus* polysaccharide attenuates renal tissue damage

**FIGURE 2:**
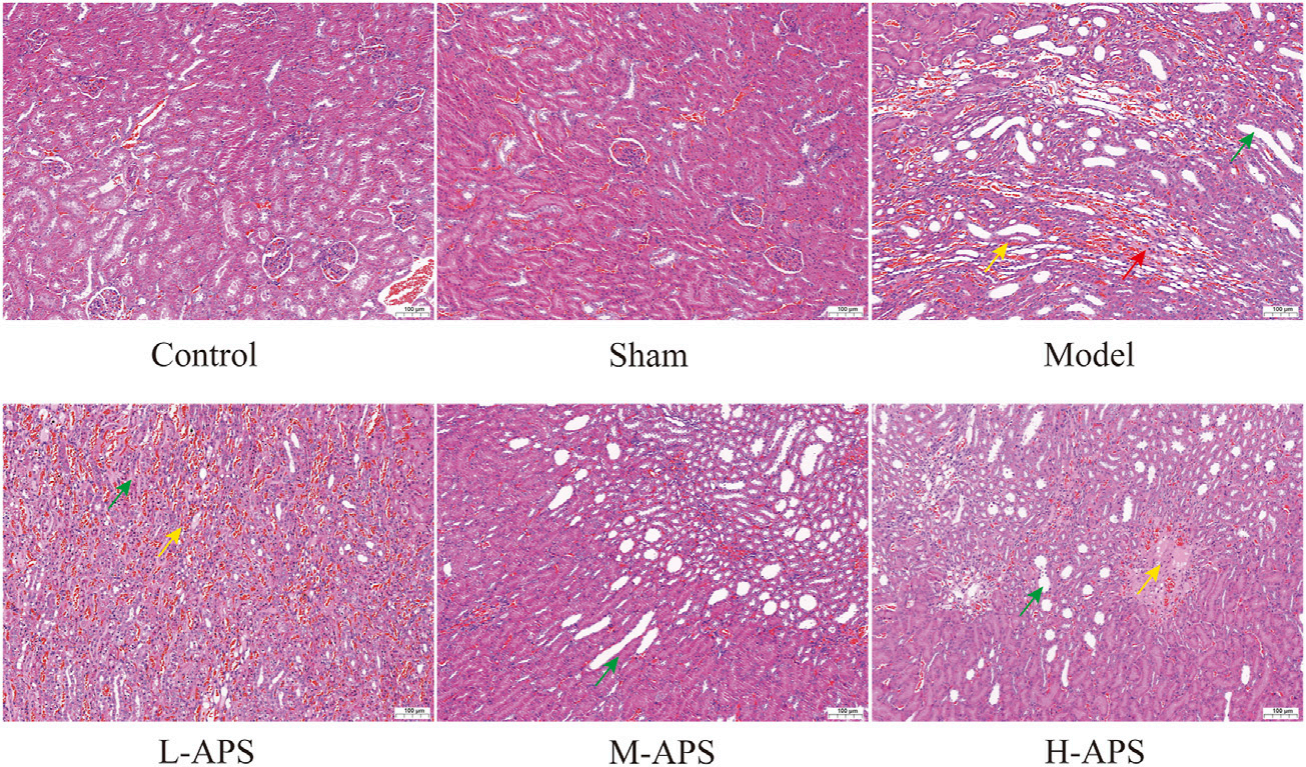
H&E staining detection of the effect of APS on renal tissue injury in septic rats. Green arrow, dilated tubules; red arrow, protein cast; yellow arrow, congestion of blood vessels. The scale bar is 100 μm.

**FIGURE 3:**
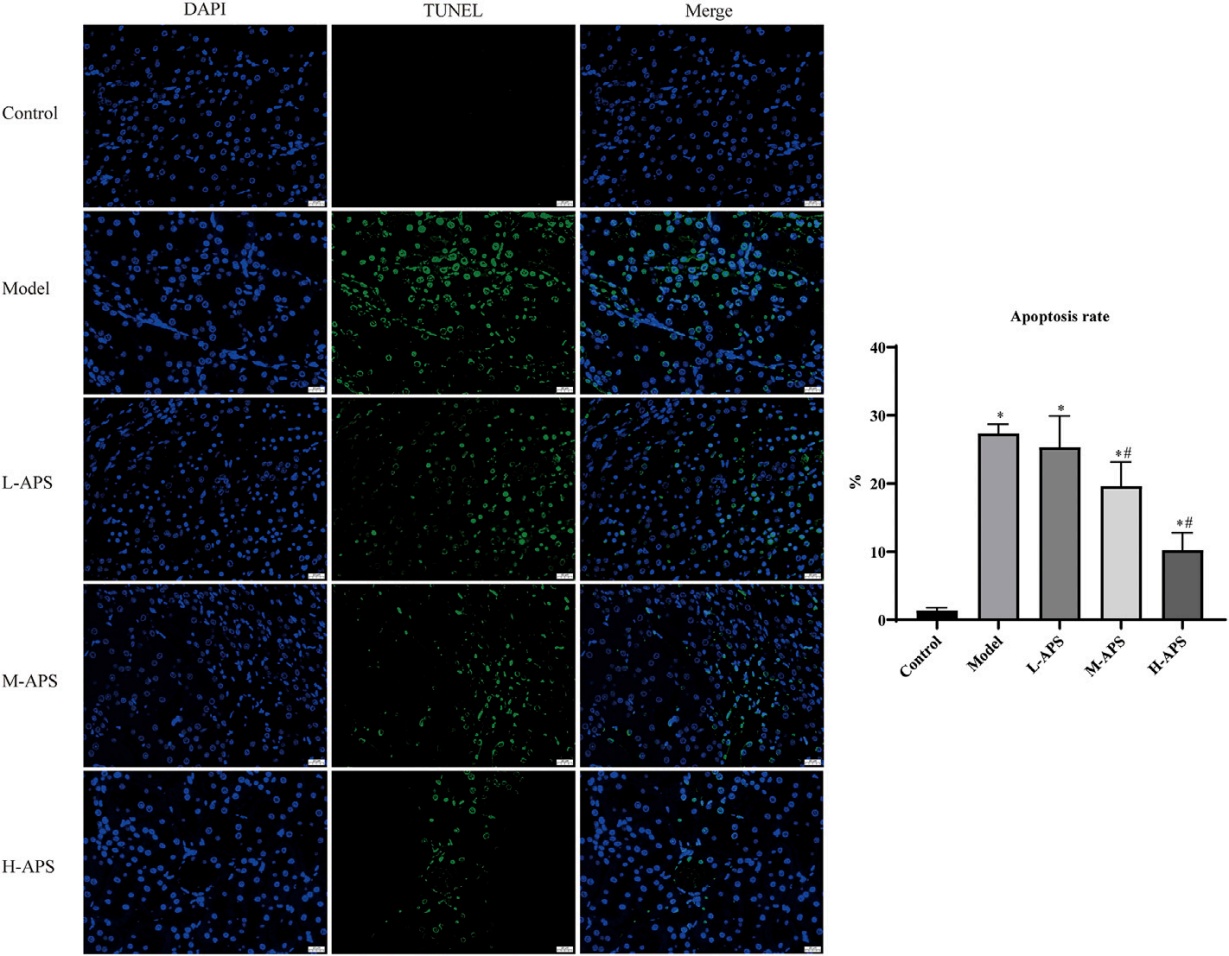
Fluorescence TUNEL staining detection of the effect of APS on renal tissue injury in septic rats. *p < 0.05 vs. control; ^#^ *p* < 0.05 vs. model. The scale bar is 20 μm.

The H&E staining results of rat kidney tissue indicated an integrated capsule of the renal tissue in control and sham groups, with a clear boundary between the cortex and medulla, a complete glomerular structure in the cortical area, and a compact arrangement of renal tubules. No obvious damage or necrosis was revealed. Except for the sham group, the renal tissues of the rats in the rest of the groups showed different degrees of damage versus control. Among them, the rat renal tubule dilation and vascular congestion in the medulla area in the model group were more obvious. With the increase in APS dosage, renal tubular dilation and vascular congestion were attenuated gradually (Figure 2). To further verify the protective effect of APS on the kidneys of septic rats, we carried out fluorescence TUNEL staining and evaluated the apoptosis of rat kidney cells. The results indicated that the number of apoptotic cells in the model group increased substantially compared to that of control, whereas that of the M- and H-APS groups greatly decreased compared with that of the model group (Figure 3).

### *Astragalus* polysaccharide reduces the expression of CYP24A1 but promotes that of CYP27B1 in rat kidneys

**FIGURE 4:**
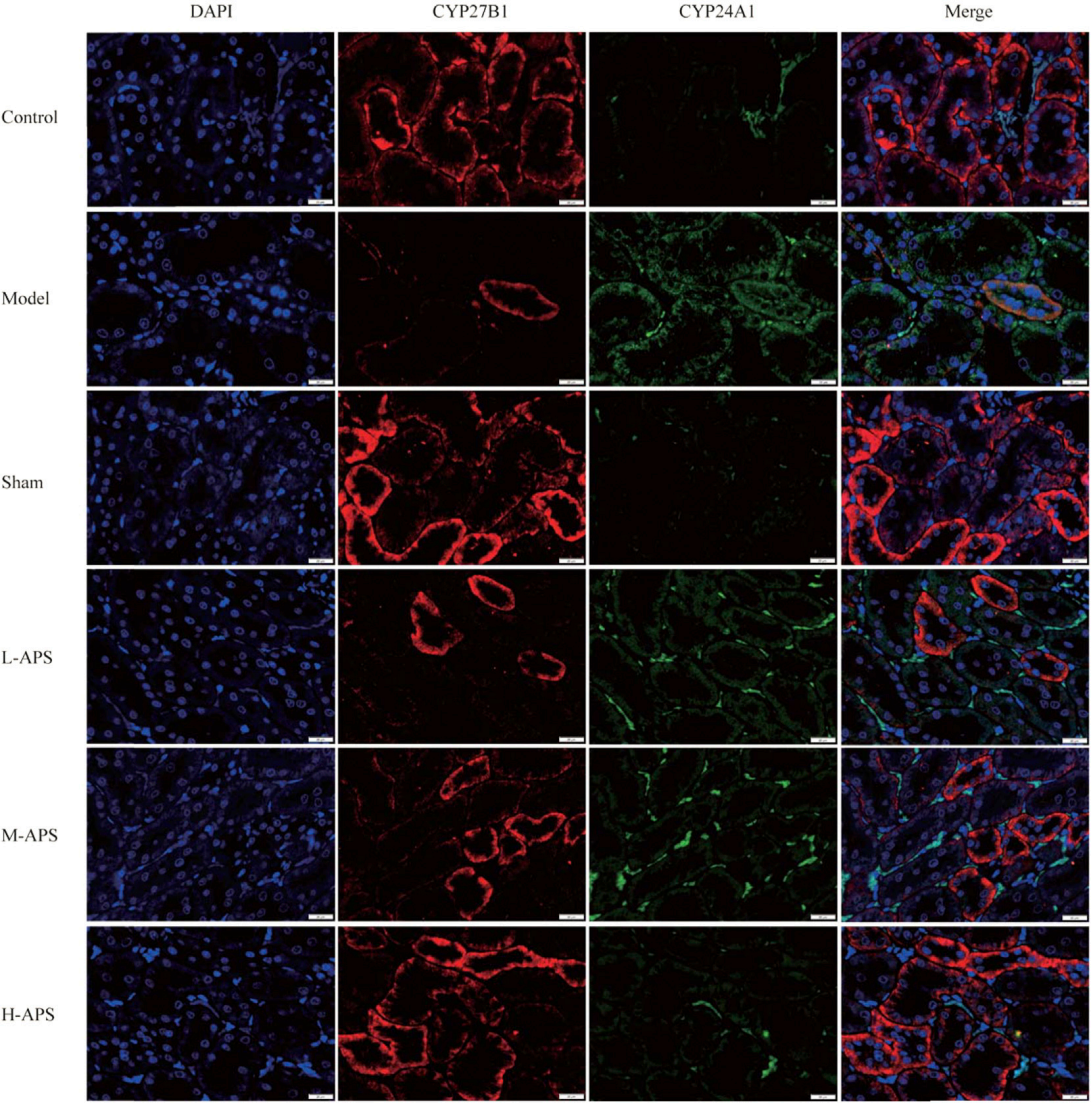
Immunofluorescence double staining detection of the protein expression of CYP27B1 and CYP24A1 in the rat kidneys. The scale bar is 20 μm.

Double immunofluorescence staining of CYP27B1 and CYP24A1 proteins in the rat kidneys indicated that the expression of CYP27B1 protein in the model group decreased whereas that of CYP24A1 increased compared to that of control. The expression of CYP27B1 of the rat kidneys in the APS groups increased with the increase of the administration concentration, while that of CYP24A1 decreased with the increase of drug concentration as compared to the model group (Figure 4).

### *Astragalus* polysaccharide reduces the expression of adrenal cortex-related proteins in septic rats

**FIGURE 5:**
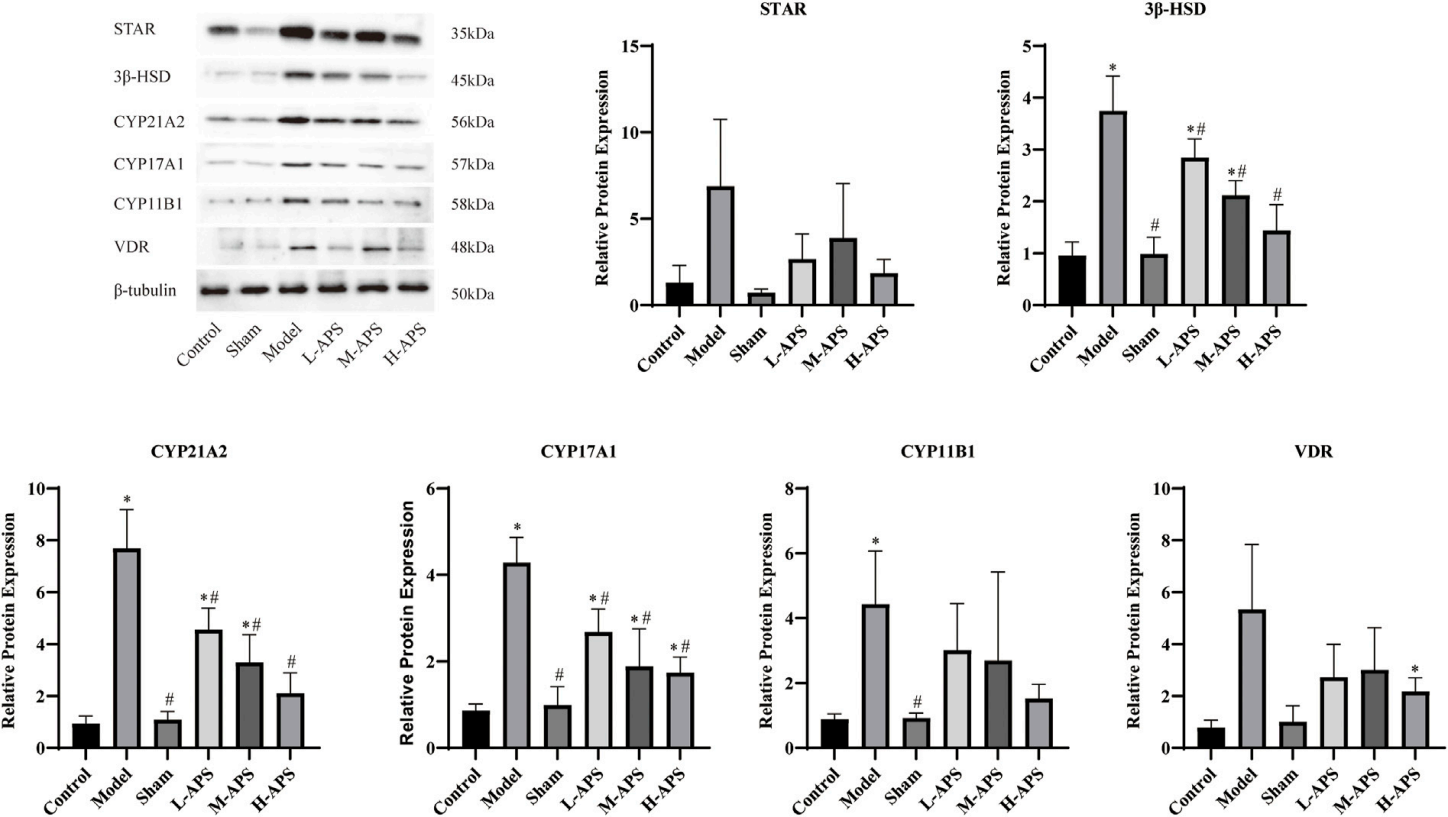
Western blotting assay detection of the effect of APS on the VDR protein surface in the adrenal cortex of septic rats. *p < 0.05 vs. control; ^#^ *p* < 0.05 vs. model.

To preliminarily explore the mechanism of APS alleviating rat sepsis, the adrenal cortex-related proteins STAR, 3β-HSD, CYP21A2, CYP17A1, CYP11B1, and VDR were detected by Western blotting. Compared to the rats in the control and sham groups, the expression of the five described proteins of the rat adrenal glands was apparently increased in the model group. The protein expression of VDR, CYP21A2, CYP17A1, and 3β-HSD in the L-APS, M-APS, and H-APS groups was markedly decreased when compared to those of the model group. The STAR, VDR, and CYP11B1 protein expressions in the rat adrenal cortex in H-APS groups were in a decreasing trend (Figure 5). The results of each group manifested that the H-APS group produced the best effect on reducing the expression of each protein.

### *Astragalus* polysaccharide increases short-chain fatty acids and the acetic acid content in the serum of septic rats

**FIGURE 6:**
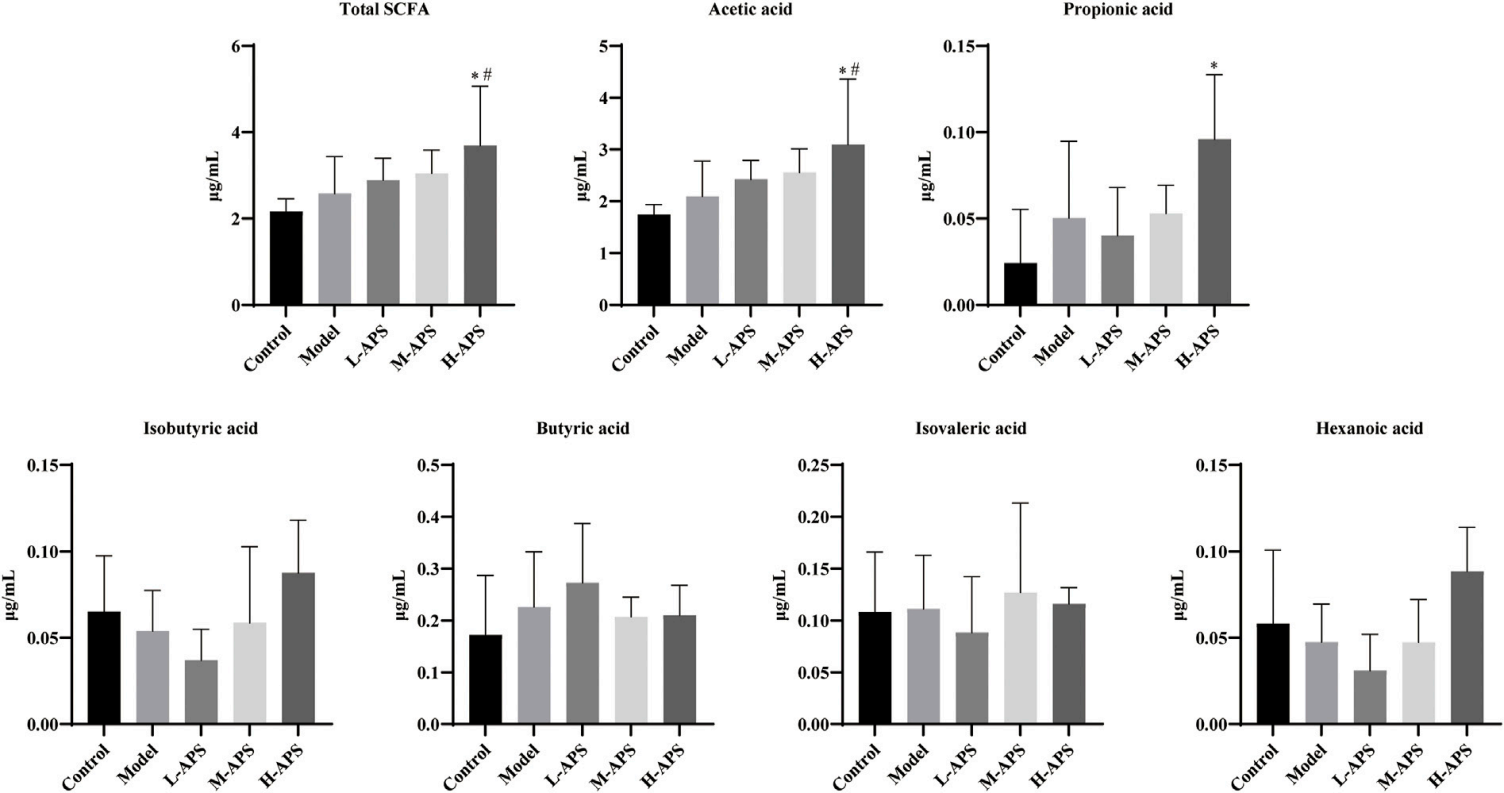
GC-MS detection of the effect of APS on the serum SCFA content. *p < 0.05 vs. control; ^#^ *p* < 0.05 vs. model.

Six types of SCFAs were detected in the rat serum using the GC-MS method. As compared to control, the serum levels of total SCFAs, acetic acid, propionic acid, butyric acid, and isovaleric acid in the model group were relatively increased, whereas those of isobutyric acid and hexanoic acid were relatively decreased. No statistical significance was revealed. As compared to the model group and control, the serum total SCFA contents and acetic acid in the H-APS group were greatly augmented. However, the contents of propionic acid, isobutyric acid, butyric acid, isovaleric acid, and hexanoic acid had no apparent difference and presented irregular changes versus model group (Figure 6).

### The effect of *Astragalus* polysaccharide on the number of intestinal flora species

The alpha diversity analysis indicated satisfactory coverage in each group. The observed OTUs and Chao1 in the groups of L-APS and M-APS were markedly higher than those in the control group (Table 1). The average number of species of the rat flora in each group is shown in Table 2. The abundance of phylum, class, order, family, genus, and species of gut microbiota was greater in the APS groups than in the control group.

**TABLE 1:** Alpha diversity index for each group.

Group	Observed OTUs	Shannon	Simpson	Chao1	Goods coverage
Control	975.83 ± 216.37	7.14 ± 0.55	0.97 ± 0.01	977.43 ± 218.28	1.00
L-APS	1171.00 ± 47.143*	7.76 ± 0.32	0.98 ± 0.01	1173.93 ± 47.96*	1.00
M-APS	1158.17 ± 121.99*	7.61 ± 0.64	0.98 ± 0.02	1159.73 ± 121.39*	1.00
H-APS	1082.67 ± 139.64	7.57 ± 0.71	0.98 ± 0.02	1084.65 ± 139.19	1.00

Note: *p < 0.05 vs. control.

**TABLE 2:** Average taxonomic abundance of rat microflora in each group.

Group	Phylum	Class	Order	Family	Genus	Species
Control	11.67 ± 0.82	19.83 ± 0.41	27.83 ± 2.14	51.00 ± 5.48	128.83 ± 18.33	165.50 ± 26.70
L-APS	11.80 ± 0.84	20.00 ± 1.22	29.00 ± 2.45	54.20 ± 2.59	143.40 ± 6.58	186.40 ± 13.13
M-APS	12.00 ± 1.26	20.17 ± 1.47	28.33 ± 1.21	55.33 ± 2.66	145.17 ± 7.94	190.33 ± 14.33
H-APS	12.00 ± 0.89	20.17 ± 0.75	27.33 ± 1.51	53.83 ± 3.06	143.50 ± 16.33	184.67 ± 24.86

**FIGURE 7:**
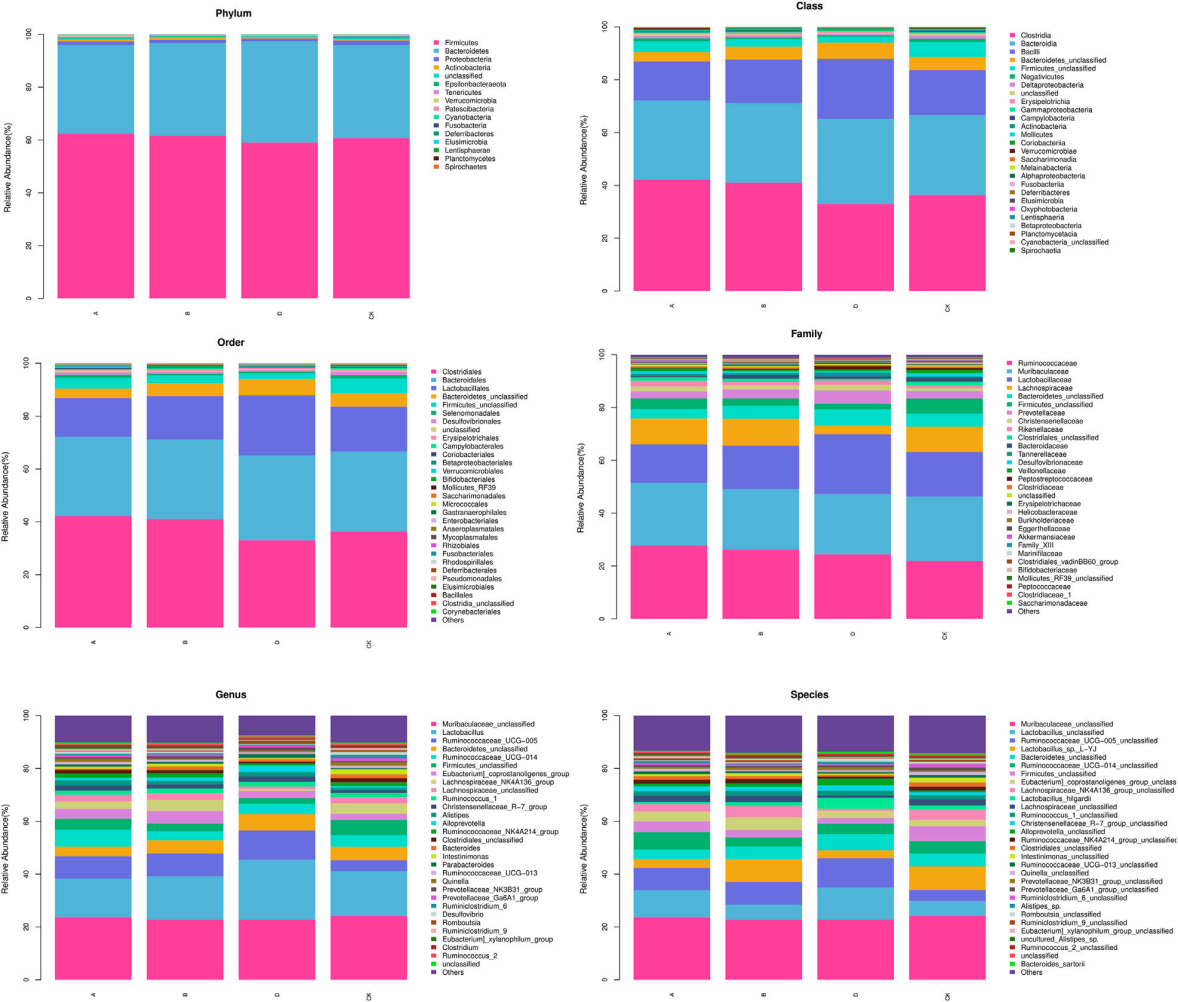
Abundance clustering of each group. A, L-APS; B, M-APS; CK, H-APS; D, control.

The detailed cluster analysis results at different levels of the intestinal flora of each group of samples are shown in Figure 7. The flora detected at the phylum level were Firmicutes, Bacteroidetes, Proteobacteria, Actinobacteria, Epsilonbacteraeota, Tenericutes, Verrucomicrobia, Patescibacteria, Cyanobacteria, Fusobacteria, Deferribacteres, Elusimicrobia, Lentisphaerae*,* Planctomycetes, and Spirochaetes. Firmicutes and Bacteroidetes accounted for the most in each group. At the class level, the top 5 included Clostridia*,* Bacterodia*,* Bacilli*,* Negativicutes*,* and Deltaproteobacteria. The top 5 flora at the order level were Clostridiales, Bacteroidales, Lactobacillales*,* Selenomonadales*,* and Desulfovibrionales. The top 5 abundance at the family level were Ruminococcaceae, Muribaculaceae, Lactobacillaceae, Lachnospiraceae, and Prevotellaceae. Prevotellaceae in the H-APS group was greatly increased versus control (*p* = 0.046). The top 3 flora abundance at the genus level were *Lactobacillus*, *Ruminococcaceae*_UCG-005, and *Ruminococcaceae*_UCG-014, and the abundance of *Ruminococcaceae*_UCG-014 in L-APS was greatly higher than control (*p* = 0.036). The most abundant flora at the species level was *Lactobacillus_*sp._L-YJ.

**FIGURE 8:**
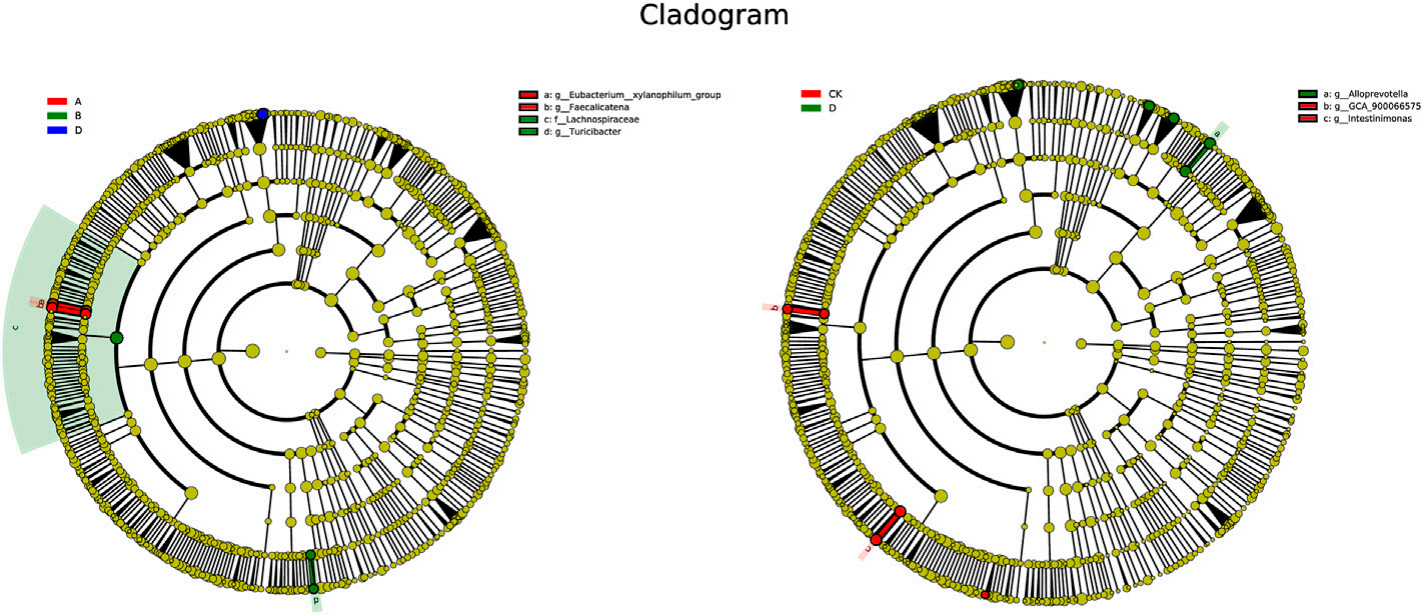
LEfSe multi-level species hierarchy tree. The nodes with different colors corresponding to the groups in the figure indicate significant enrichment in this group, and the microbial community has a significant impact on the differences between groups. The nodes marked in yellow indicate the microbe groups that have no significant difference among different groups. A, L-APS; B, M-APS; CK, H-APS; D, control.

As compared to control, the abundance of g_*Eubacterium_xylanophilum*_group and g_*Faecalicatena* was higher in the L-APS group at the genus level. The abundance of f_*Lachnospiraceae* and g_*Turicibacter* in the M-APS group were higher. The abundance of g_GCA_900066575 and g_*Intestinimonas* were higher in the H-APS group (Figure 8). The previously described results suggested that appropriate APS dosage could increase the abundance of intestinal flora species, thereby improving the structure and function of intestinal flora in rats.

## Discussion

Sepsis represents organ dysfunction, which arises from a maladjusted body response to an infectious agent, and the most common organ damage is acute kidney injury (AKI), an occurrence of up to 50% among patients (Manrique-Caballero et al., 2021). A more serious consequence of kidney damage is a direct reduction in active vitamin D (VD) generation. VD is recognized as a class of steroid derivatives necessary to maintain the life of higher animals, and its most functional active form in the body is 1,25-dihydroxy vitamin D3 [1,25(OH)_2_D_3_]. VD_3_ is a precursor of 1,25(OH)_2_D_3_ and is the main form of VD present in the human body. VD_3_ passes through the liver and is hydroxylated to 25-hydroxyvitamin D3 [25(OH)D_3_]. Due to 25(OH)D_3_ having a long half-life period, its serum level is usually applied to monitor the VD content in the body (Holick, 2005). 25(OH)D_3_ in the blood is captured by VD-binding protein and transported to the kidney, where 1,25(OH)_2_D_3_ is formed by 1α-hydroxylase (CYP27B1) in the renal mitochondria, thereby exerting biological effects *via* blood flow transportation and binding to VD receptor (VDR) on target organs (Priyadarshini et al., 2021). In addition to CYP27B1, 24-hydroxylase (CYP24A1) is highly expressed in the kidney as well, which can inhibit 25(OH)_2_D_3_ activation and metabolize 1,25(OH)_2_D_3_, i.e., CYP27B1 and CYP24A1 are a key protease pair that can maintain 1,25 (OH)_2_D_3_ at normal levels (Jones et al., 2012). The VD level of patients was strongly negatively correlated with disease severity and mortality, and the probability of sepsis and acute renal failure in patients with VD deficiency was higher than that in healthy people (Parekh et al., 2017). VD supplementation can reduce the levels of TNF-α and IL-6, improve the coagulation parameters of disseminated intravascular coagulation, and enhance the induction of cathelicidin and B defensin in animal models of sepsis, thereby restricting the inflammatory attack of sepsis on multiple organs throughout the body, including the adrenal cortex (Olejarova et al., 2019). Moreover, VD can significantly increase the mRNA expression of STAR, CYP17A1, and CYP21 proteases in adrenal cortex cells, and intragastric administration of VD to sepsis model rats can increase the serum cortisol level *in vivo* (Jeremy et al., 2019). Cortisol is a major component of glucocorticoid, which is transferred from cholesterol through STAR, 3β-HSD, CYP21A2, CYP17A1, and CYP11B1 on the adrenal cortex. It exerts a key role in maintaining system homeostasis, including anti-inflammatory, anti-shock, anti-toxin, and metabolic regulation effects; improving excitability; and stabilizing blood pressure and blood sugar in the central nervous system. Clinical studies have shown that 60% of sepsis patients are accompanied by hypercortisolemia in early sepsis (Flierl et al., 2009), which can effectively reflect the occurrence of inflammation in sepsis patients.

This study indicated that the content of VD_3_ showed an increasing trend with the incremental APS dosage, but 25(OH)D_3_ and 1,25(OH)_2_D_3_ contents in the H-APS group were greatly increased compared to those in the model group. Meanwhile, HE staining and TUNEL tests revealed that APS could alleviate the renal tubular dilation and vascular congestion and markedly reduce the number of apoptosis in the kidney tissues of rats. Immunofluorescence results indicated that APS increased CYP27B1 protein expression and decreased CYP24A1 in rat kidneys, indicating that APS could effectively prevent renal cell apoptosis, reduce protein cast formation, and inhibit the occurrence of renal injury in rats with sepsis. Increasing the contents of the three previously described vitamin Ds in the body and correcting the protein expression of CYP27B1/CYP24A1 of the kidneys could greatly increase the 1,25(OH)_2_D_3_ content, thereby protecting the overall activity level of the VD axis and minimizing the renal damage caused by sepsis.

C-reactive protein (CRP) belongs to an innate immune system synthesized by the liver acute-phase reactive protein. After being combined with the ligand phosphorylcholine, it can identify pathogens and activate phagocytosis by initiating the classical complement system or the C1q pathway of the serum complement (Eschborn and Weitkamp, 2019). The CRP concentration in the serum increases rapidly when the body tissue is infected or injured, and it is the most sensitive indicator of inflammation (Del Giudice and Gangestad, 2018). Under inflammation, the expression of soluble intercellular adhesion molecule-1 (sICAM-1) *in vivo* or abscission from the cell surface increases, resulting in an elevated content of sICAM-1 in the blood. Hence, it is an important index to evaluate the activity of this disease. sICAM-1, which is widely distributed in the vascular endothelium, can adhere to ligand lymphocyte function-associated antigen 1 (LFA-1) and carry rapidly moving inflammatory cells from blood vessels to peripheral inflammatory tissue lesions (Kapetanovic et al., 2021). The binding of free sICAM-1 to LFA-1 can promote the killing process involving CTL and NK cells, enhance the immune response of helper T cells, and involve in antibody-dependent cell-mediated cytotoxicity (ADCC) (Gross et al., 2010). Sun et al. (2021) showed that APS inhibited the expression of TNF-α, IL-1 β, IL-6, and IL-8 in a concentration-dependent manner to reverse sepsis-induced acute kidney injury in mice. Dong et al. (2019b) demonstrated that APS reduced the mRNA expression levels of TNF-α, IL-1 β, IL-6, and IL-17 in diarrheic mice. Our results also revealed that APS exerted its anti-inflammatory effect by decreasing the content of TNF-α, IL-6, CRP, sICAM-1, and CORT in the serum. Also, APS reduced the protein expressions of VDR, STAR, 3β-HSD, CYP21A2, CYP17A1, and CYP11B1 in the adrenal cortex of rats, suggesting that APS inhibited the transformation of STAR, 3β-HSD, CYP21A2, CYP17A1, and CYP11B1 to CORT. The present results proved that APS prevented the increase of CORT in the serum and alleviated the inflammatory response caused by sepsis.

Intestinal flora refers to the complex and enormous micro-ecosystems parasitic in the human intestine. In addition to promoting intestinal digestion, absorption, and excretion, short-chain fatty acids (SCFAs) and their metabolites can also complete physiological and biochemical functions, namely, energy metabolism, immune defense, and vitamin synthesis, by parasitizing the host. The occurrence of sepsis will destroy the homeostasis of intestinal flora, resulting in decreased biodiversity of the micro-ecosystem, increased number of pathogenic bacteria, low SCFA concentration, and probiotic proportion (Fay et al., 2019). It has been proven that changes in the intestinal microbial communities can affect the susceptibility to inflammation in mice (Hayakawa et al., 2011). Imbalanced flora will promote the production of intestinal endotoxin. By passing through the intestinal mucosal barrier with increased permeability, the endotoxin reaches various organs and tissues outside the intestinal tract with blood flow, facilitating the release of pro-inflammatory factors, inducing systemic inflammatory response syndrome, and accelerating the occurrence of multiple organ damage (Xu et al., 2019). APS could decrease the Firmicutes to Bacteroidetes ratio and increase the abundance of Proteobacteria and Epsilonbacteria to reshape the intestinal microbiome in non-alcoholic fatty liver disease rats (Zhong et al., 2022). In diarrheic mice, APS significantly increased the numbers of *Lactobacillus* and *Bifidobacterium* spp*.* to normal levels (Dong et al., 2019b). However, how APS changes the intestinal flora remains unclear. APS is a kind of complex mixture composed of arabinose, mannose, and xylose (Chen et al., 2015; Zheng et al., 2020). APS is difficult to be decomposed into monosaccharides after entering the digestive tract. Meanwhile, the absorption rate of APS in intestinal epithelial cells into the blood is extremely low, so it can hardly interfere with the internal environment of the body directly. Therefore, it is important to understand the mechanism of its biological activity to reveal the mechanism of its prevention and treatment of sepsis. Studies have found that polysaccharides are hydrolyzed into SCFAs and release adenosine triphosphate through a series of complex pathways such as glycolysis and pentose phosphate under the action of microorganisms in the gut. Subsequently, the intestinal tract takes advantage of the energy from SCFAs and exchanges SCFA into the blood in the form of anions (Tan et al., 2014; Krishnan et al., 2018). The present study illustrated that the total concentration of SCFAs in the serum of septic rats was increased versus control, and APS could effectively increase the content of SCFAs, especially acetic acid, in septic rats. However, it had no apparent effect on the other SCFA levels in the serum. Acetic acid has indicated a significant role in the progression of sepsis. At the onset of sepsis, the intestinal flora may correct the disturbed immune system by increasing SCFA generation, while APS can interfere with the inflammatory network effect and prevent organ inflammatory injury by promoting intestinal flora.

Taken together, APS can effectively antagonize the pathological damage to the adrenal cortex caused by sepsis, alleviate tissue inflammation, stimulate the expression of adrenal STAR, CYP21, CYP17A1, and other active enzymes that have the effect of promoting the synthesis of cortisol, and increase cortisol levels in the serum. Moreover, the level of 25(OH)D_3_ and the expression of VDR in model rats can be corrected. It is speculated that APS may protect the tissue structure of the adrenal cortex and its function of synthesizing and secreting cortisol by regulating the activity level of the vitamin D axis, so as to play a certain role in sepsis treatment, but its mechanism of action at the molecular level needs to be further elucidated. In addition, APS can enhance the abundance of gut microbiota and improve the structure and performance of the flora. Upregulation of the total SCFA content and acetic acid content in septic rats suggested that after APS entered the body, it was converted into acetic acid, entered into the blood by the intestinal flora, and played a certain role in this disease. However, its active form of entering the body and its regulatory effect on the VD axis need further investigation and verification.

## Conclusion

APS could upregulate the VD level and adrenocortical VDR content in septic rats using CLP, reduce the inflammatory response in rats and cortisol levels in the serum, and increase the rat intestinal flora abundance, thereby promoting SCFAs and the acetic acid content in septic rats to jointly reduce the sepsis reaction and renal damage caused by sepsis.

## Data Availability

The original contributions presented in the study are included in the article/Supplementary Material; further inquiries can be directed to the corresponding author.
